# Insights into the Evolution and Emergence of a Novel Infectious Disease

**DOI:** 10.1371/journal.pcbi.1000947

**Published:** 2010-09-30

**Authors:** Ruben J. Kubiak, Nimalan Arinaminpathy, Angela R. McLean

**Affiliations:** Institute for Emerging Infections, James Martin 21st Century School, Department of Zoology, University of Oxford, Oxford, UK; University of New South Wales, Australia

## Abstract

Many zoonotic, novel infectious diseases in humans appear as sporadic infections with spatially and temporally restricted outbreaks, as seen with influenza A(H5N1). Adaptation is often a key factor for successfully establishing sustained human-to-human transmission. Here we use simple mathematical models to describe different adaptation scenarios with particular reference to spatial heterogeneity within the human population. We present analytical expressions for the probability of emergence per introduction, as well as the waiting time to a successful emergence event. Furthermore, we derive general analytical results for the statistical properties of emergence events, including the probability distribution of outbreak sizes. We compare our analytical results with a stochastic model, which has previously been studied computationally. Our results suggest that, for typical connection strengths between communities, spatial heterogeneity has only a weak effect on outbreak size distributions, and on the risk of emergence per introduction. For example, if 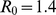 or larger, any village connected to a large city by just ten commuters a day is, effectively, just a part of the city when considering the chances of emergence and the outbreak size distribution. We present empirical data on commuting patterns and show that the vast majority of communities for which such data are available are at least this well interconnected. For plausible parameter ranges, the effects of spatial heterogeneity are likely to be dominated by the evolutionary biology of host adaptation. We conclude by discussing implications for surveillance and control of emerging infections.

## Author Summary

Emerging infections are a continuing global public health issue, the most recent example being last year's ‘Swine flu’ influenza pandemic. However, for many zoonotic pathogens, some adaptation is required to cross the species barrier from an animal reservoir into humans and cause sustained transmission. Previous work has explored the relationship between the evolutionary biology of an adapting pathogen, and the epidemiology of cases that may arise before such a pathogen becomes pandemic-capable. Here, we extend this work to incorporate what is often an important host ecological feature, the spatial distribution of the host population. Many zoonoses occur away from large population centres. For example, HIV is thought to have entered the human population through bushmeat hunters in the sparsely populated jungles of Central Africa. We ask: when a pathogen is evolving to adapt for human transmission, under what circumstances does the spatial structure underlying the human population become important? We approach this question using mathematical models to explore regimes of connectedness between communities. Our results suggest that most communities are sufficiently interconnected to show no effect on the emergence process. We finish by discussing the implications of these findings for public health.

## Introduction

Zoonotic emergence of novel human infections poses a significant risk to global public health. For example, the ‘Spanish flu’ pandemic of 1918 probably originated in birds and caused millions of deaths worldwide [1]. While much less virulent, the subsequent influenza pandemics of 1957, 1968 and 2009 [2], [3] are potent reminders of the capacity of the influenza virus to cross the species barrier into humans. Many other pathogens share this capacity: the SARS outbreak of 2003 [4]–[6] has been linked to bats and palm civets [7], [8]. In 2008, a novel arenavirus which killed four out of five patients in South Africa was linked to rodents [9].

Previous work [10], [11] has studied models of within-host evolution and between-host transmission, in which an initially poorly transmitting pathogen acquires adaptations to human hosts, following repeated zoonotic introductions until it achieves pandemic potential. These make the natural, simplifying assumption that the host population is homogeneous, so that changes in infection parameters entirely reflect adaptations in the biology of host infection. In reality, however, factors such as human contact patterns [12] and other host heterogeneity [13], [14] may also shape the risk and speed of emergence events. We concentrate here on the heterogeneity in the spatial structure of the human host population, an area which has hitherto received little attention in the context of adapting pathogens. We model spatially separated communities with varying types and strengths of interconnections, for example between a village and a city. Our aim is to study under what regimes such ‘ecological’ structure could have a strong effect on emergence, in comparison with ‘evolutionary’ factors governing the biology of infection.

In the following section we give an overview of the modelling approach. We then present new analytical results for the simple models studied previously, which ignored spatial host population structure. We use these expressions to provide useful answers to important questions: if we knew how a zoonotic pathogen would adapt to human physiology, could we anticipate its emergence? How reliable would such predictions be? Furthermore, can we predict which zoonoses will cause outbreaks which do not turn into epidemics? Next, we ask: how large does a single, finite host population have to be, for population size to have a negligible effect? We then incorporate spatial heterogeneity by separating the human host population into communities. We present a model in which a small village is connected, by human travel, with a large city as an example of the general case of two interconnected communities. We use this model to ask: how strong do community interconnections have to be for us to safely ignore the separation of a population into spatially structured communities, such as cities and villages? We review available commuting data to ask how these thresholds compare with typical human mobility patterns? We close with a discussion of implications for public health.

## Materials and Methods

### Modelling Evolutionary Adaptation

We build on a model of evolution and emergence originally presented by [10] in which a zoonotic pathogen infects humans, and initially has very poor onward transmissability. Thus for people who are infected by animals the average reproductive number, 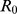, is well below one (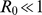). We call this the first reproductive number 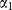 for the wildtype strain. Occasionally, during such zoonotic infections, the pathogen acquires genetic changes that increase its ability to pass to other humans. During any chain of transmission the pathogen might adapt sufficiently that it achieves such ease of human-to-human transmission that 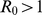 and an epidemic becomes possible. Such a process can be characterised by a vector of reproductive numbers (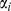 with 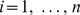), and a vector of mutation probabilities (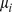 with 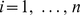) where 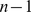 denotes the number of adaptive steps necessary to reach the fully adapted strain 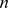.

In what follows we restrict our attention to the case of 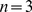, 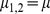 and 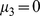, allowing us to model two routes of adaptation with opposite and distinct characteristics while minimising the overall complexity in number of required strains. For both routes of adaptation, the first, wildtype strain has very low transmissibility, the third has pandemic potential, and the second strain has intermediate transmissibility. This intermediate transmissibility is not enough to sustain the novel pathogen within the human host population, but secondary infections by humans are possible. Thus we have 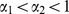. Finally, the human adapted strain has a reproductive number 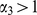. Further, we assume an identical mean infectious period for all strains.

Following [11], we first distinguish two routes to adaptation: the ‘punctuated’ scenario has an evolutionary course 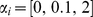, while the ‘gradual’ scenario has 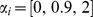, the only difference being 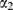, the fitness of the intermediate stage.

This leads to the following SIR-model, normalized with respect to the mean infectious period,
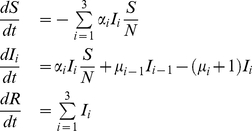
(1)where 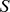 is the number susceptible, 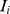 is the number infected with strain 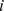, and 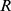 the number of recovered or removed. We do not include births and deaths as we expect a zoonotic emergence, or extinction, to happen on a much shorter timescale than the human lifespan. We translate this model to a stochastic simulation of a multitype branching process, using the Gillespie algorithm [15]. The infection is seeded in a single random, susceptible host with the wildtype strain.

In general, every introduction has only two possible outcomes: emergence or extinction. Extinction happens if the novel pathogen dies out because it fails to adapt for human transmission or just by stochastic extinction. Hence, the introduction only leads to a limited number of infectious hosts, which we refer to as the ‘outbreak size’.

Conversely, a novel pathogen of zoonotic origin emerges if it is sufficiently adapted for human transmission and begins to spread in a self-sustaining way. Formally, in an unlimited host population the cumulative number of infectious hosts is unbounded as time goes to infinity. Computationally we use a threshold of 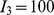 infectious hosts with the fully adapted strain to distinguish between emergence and extinction. This threshold ensures a probability of extinction less than 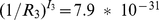 [16]. Therefore, the number of falsely identified emergences, which would truly be extinctions, is negligibly small. Moreover, these arbitrarily small probabilities ensure that our simulation results are insensitive to the precise choice of threshold used. In situations where the host population is very small we relax our emergence threshold to smaller numbers of infectious hosts as some population sizes are below 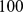.

### Probability of Emergence

In the special case 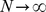 and 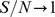 it is possible to calculate the probability of emergence per introduction into the human host population, given the evolutionary course of the pathogen, and the mutation rate with which the pathogen adapts. Our derivations start with assuming one homogeneous human host population of infinite size. This assumption can be easily relaxed as we show later. To calculate the probability of emergence, we define next event probabilities of infection 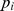, mutation 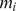, and recovery 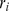 for each individual infected host, therefore the probabilities for what type of event will come next for each infectious host are
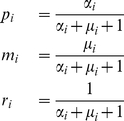
(2)


Note that in general, we can extend this adaptation process to arbitrarily many adaptive steps. The mutations are uni-directional towards the adapted strain. Using a branching-process approach similar to [10], we derive the probability of emergence per introduction as follows (see Text S1, A.1.1, for more details)
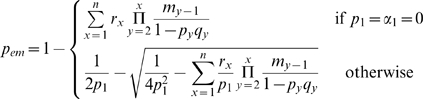
(3)The probability of emergence can be expressed by the next event probabilities and the probability of extinction 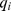 given an index infection of strain 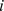. This expression can be solved analytically for all possible routes of adaptation.

### Waiting Time to Emergence

Regardless of the underlying population structure and the pathogen's biology, we can make an estimate of the number of introductions needed before an emergence arises 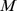, given the probability of emergence per introduction 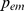 with 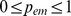 (see Text S1, A.1.3, for more details)
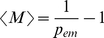
(4)Note that this is the average number of introductions without an emergence. The average number of introductions needed for an emergence event is 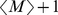.

In addition, the variance can be obtained in a similar way (see Text S1, A.1.3)
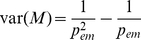
(5)This variance leads to a standard deviation of the same order as the average number of introductions, 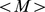. This makes the number of introductions before an emergence inherently unpredictable if the probability of emergence per introduction is small (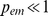).

### Outbreak Size Distribution

Again, in the special case 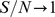, the branching-process approach can be extended to derive the probabilities of outbreak sizes before emergence (see Text S1, A.1.2, for more details). In general, the probability of an outbreak of size 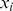 is defined as 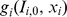 with 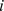 denoting the strain. The number of infected hosts to start with is denoted by 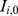. Furthermore, the overall outbreak size probability can be derived using conditional probabilities
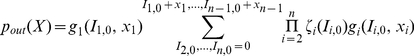
(6)where 
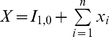
 is the total outbreak size, and 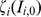 is the probability of getting 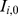 patient zeros to start with in strain 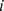. The summation in the derivation of the overall outbreak size probability is over all possible subsets of infectious hosts to start with.

### Incorporating Spatial Heterogeneity

We use a metapopulation model to explore the effects of spatial host heterogeneity, effectively dividing the human population into interconnected communities. As an example of the general case of spatially structured communities, we focus on a simple village - city model to approximate the spatial host heterogeneity in rural areas connected, by human mobility, to bigger cities. There are many different types of human mobility between communities such as villages and cities, including short-term commuting and long-term labour migration. Particularly in developing countries, however, information on dominant patterns is sparse. Nonetheless, anecdotal evidence from Vietnam [17], for example, suggests that short term commuting plays an important role: here, a subset of the village residents collects agricultural produce for trading in local markets in the city, and travels to the city on a daily to weekly basis. Accordingly we present a model in which the residence time of villagers in the city is typically less than the infectious period. However, in the supplementary information we also present a model incorporating migration on longer timescales (see Text S1, A.2). These two different models illustrate that our results appear qualitatively robust to different types of human movement.

**Figure 1:**
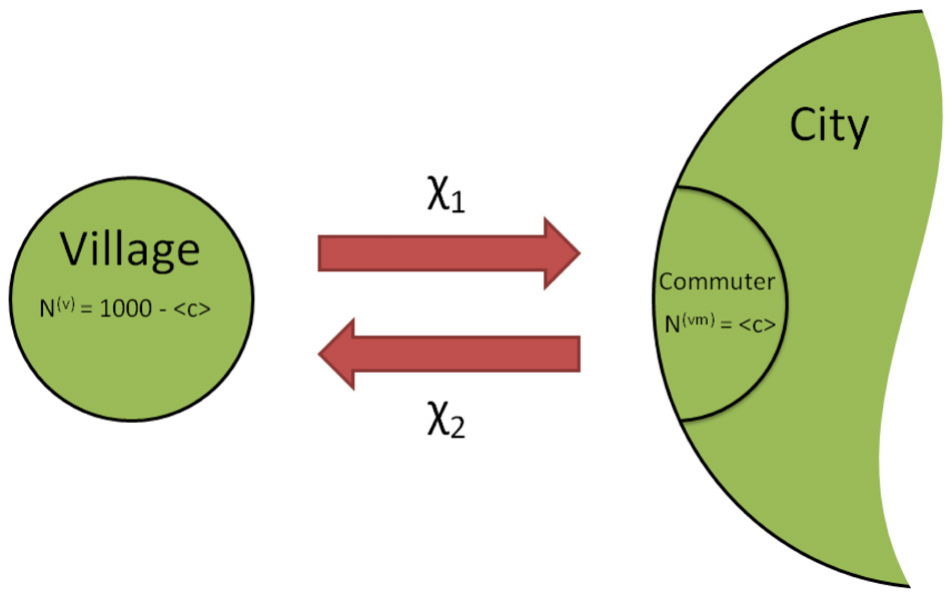
Schematic representation of the short-term commuting model. 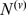
 is the number of residents present in the village and 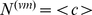 the number of commuters in the city. The city has an infinite number of residents. 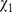 is the per capita commuting rate from the village to the city and 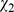 the return rate. Together, both determine the number of commuters 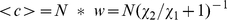.

As before, we have a wildtype pathogen capable of acquiring adaptations for human transmission. Assume a finite number of hosts in the village, and an effectively infinite number in the city. To allow for daily commuting, we label individuals in the city according to whether they are commuters from the village or not (neglecting commuters originating from the city and present in the village). The superscripts 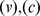 represent village and city inhabitants respectively, while 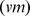 denotes villagers in the city. Village members commute to the city at a per capita rate 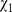, and return at per capita rate 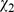. At any one time, a proportion 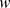 of villagers, the commuters, are in the city with 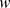 being set by 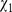 and 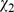 as described below. Further, we neglect susceptible village commuters acquiring infection in the city (this arises formally from the infinite number of hosts in the city). The number of village residents is fixed at 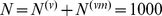, and we define the average number of commuters as
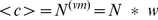
(7)
Figure 1 shows a schematic representation of this commuting model. Normalising time with respect to the mean infectious period, the governing equations are
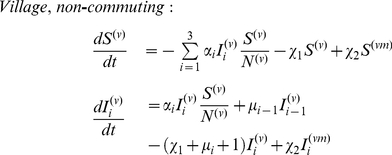
(8)

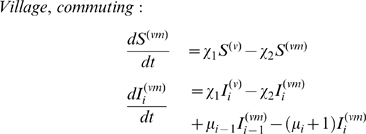
(9)

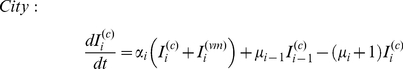
(10)

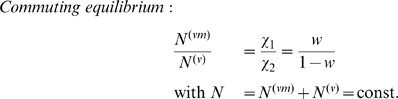
(11)Note that equation (11) arises from the fact that 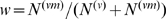. To represent daily commuting, with an infectious period of 5 days, we set 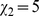, and choose 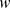 to give the required average number of commuters 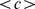. To seed a wildtype infection in the village we set 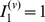. In the village-city model, an emergence event is defined as having 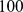 infectious hosts with the fully adapted strain in the city.

## Results

### Validation of Analytical Results

**Figure 2:**
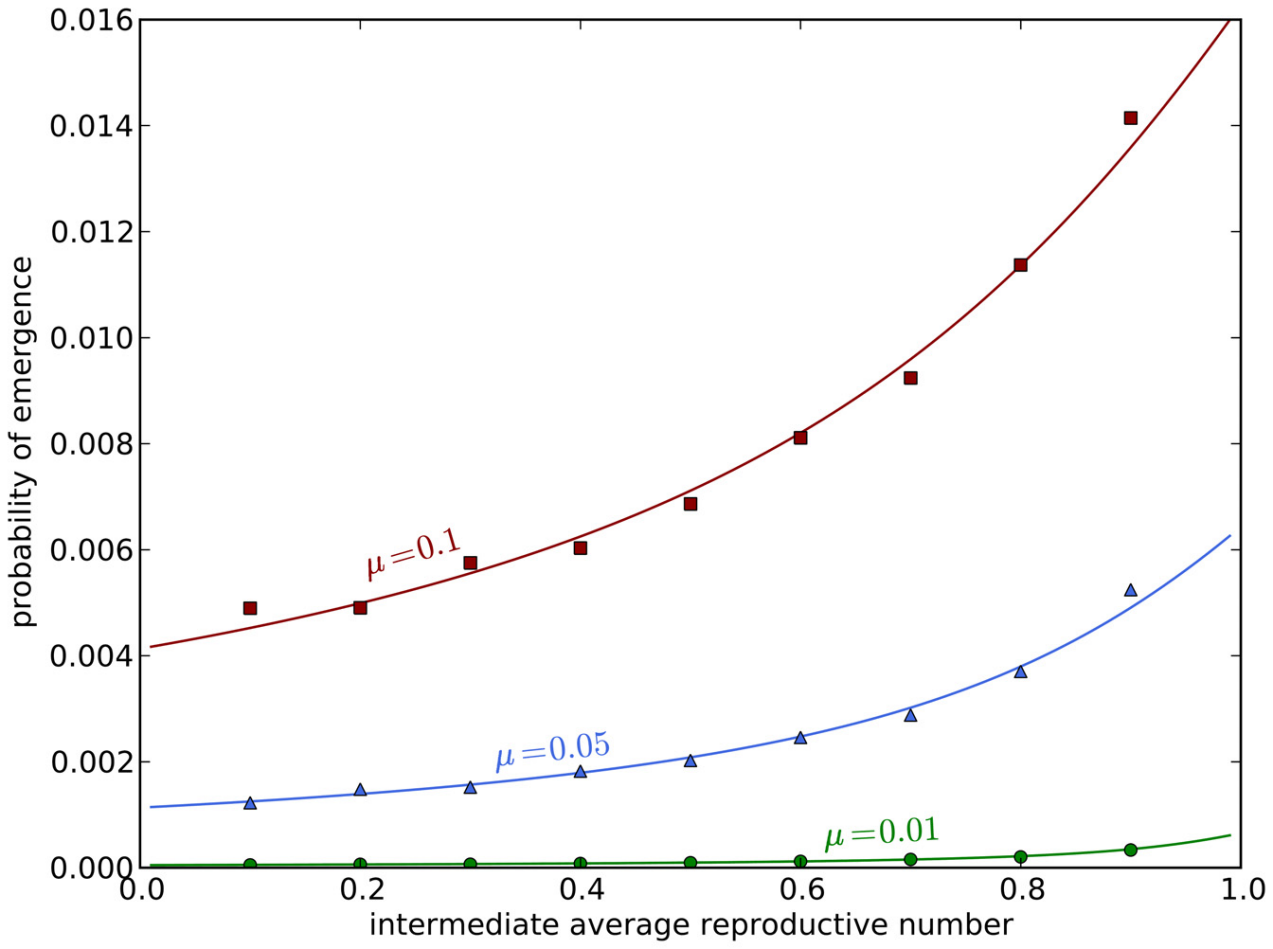
Comparison of the probability of emergence per introduction. Shown is the probability of emergence as a function of the mutation rate 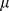 and the intermediate reproductive number 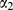. The average reproductive number for each strain is 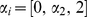 and the mutation rate 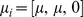, 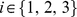. The solid lines are analytical results. The data points represent the average probability of emergence over 1000 simulated emergences with a host population size of 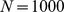. The agreement between analytical calculations and simulations is excellent. Further, the probability of emergence grows non-linearly with 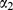 and 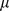.

We use the three strain model described before to study the impact of the mutation rate, 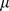, and the average reproductive number of the intermediate strain, 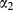, on the probability of emergence per introduction in a single, infinite population. We assume 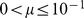 as an illustrative spectrum of possible mutation rates. Figure 2 shows the probability of emergence for different mutation rates and average reproductive numbers of the intermediate strain. Not surprisingly, the probability of emergence grows non-linearly with 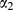 and 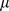. The probability of having no mutation in the second strain is 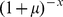 where 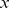 is the number of infected hosts with strain 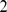. While the intermediate reproductive number affects the exponent, the mutation rate has a direct influence on the base. We validate our analytical results by comparing them with the average probability of emergence of 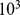 simulated emergence processes, using one homogeneous population as described in (1). Figure 2 reveals an excellent agreement between the analytical results and simulations.

### Effect of Limited Host Population Size

**Figure 3:**
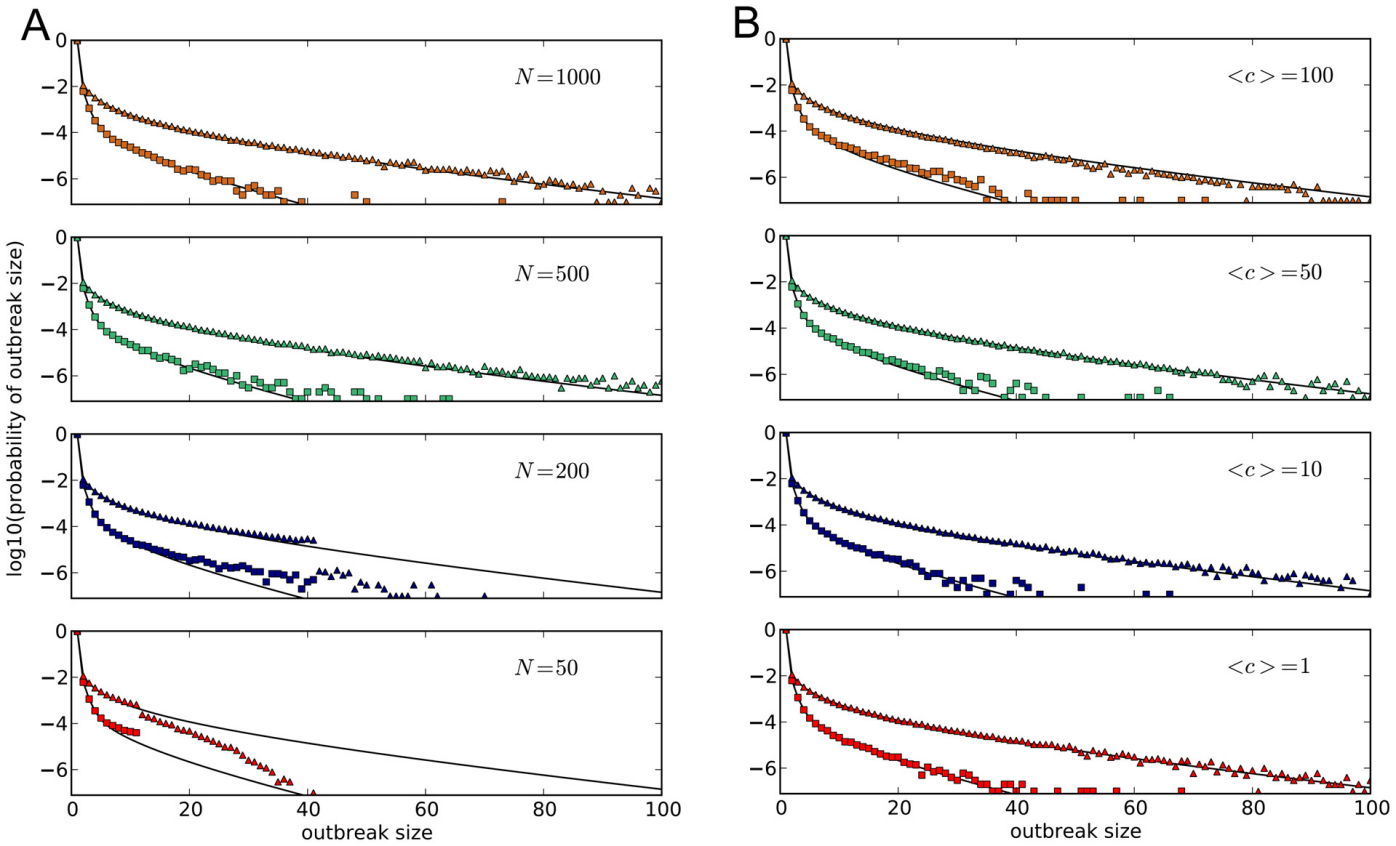
Outbreak size distribution. The solid black line represents the analytical result for an infinite population. Data points are the average probability of 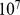 simulations. Squares represent the punctuated route (I), and triangles the gradual route (II), both with the mutation probability 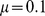. **A** Outbreak size distribution as a function of host population size. For a population size, 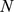, of 50, 200 and 500, an emergence event is defined as having at least 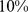 of the population size infected with the fully adapted strain, while the number was fixed at 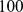 infected with the fully adapted strain for 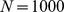. While host populations with 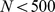 clearly show finite size effects, even small host populations with 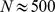 can be effectively treated as infinite as the outbreak size is small compared to the population size. The figure reveals the effect of the pathogen's evolution on the outbreak size distribution as the distributions group according to the route of adaptation. **B** Probability of emergence in the city with short-term commuting. The probability is a function of the overall number of infectious hosts in the village-city model with 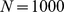. The red color represents 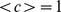, blue 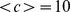, green 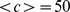 and gold 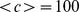 commuters. As for homogeneous populations, the outbreak size distributions group according to the evolutionary route of adaptation. Spatial heterogeneity does not have an influence as all simulations do not show a significant variation from the analytical results.

For small host communities, the depletion of susceptible hosts can play a significant role in limiting an ongoing outbreak. What is the effect of a finite population size on these analytical results which assume an infinite host population? Figure 3A compares the simulated outbreak size distribution of different sized populations with our analytical predictions. Note that, for populations greater than 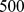, there is close agreement between numerical and analytical results. When considering populations of size 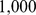 or more, we do not expect population size dependence to have a substantial effect.

### Effect of Spatial Heterogeneity

The village's number of residents in our commuting model is sufficient to avoid finite size effects on the outbreak size. Furthermore, it is independent of any spatial heterogeneity. The number of residents only has a limiting effect on the outbreak size distribution. Figure 3B shows the outbreak size distribution for our short-term commuting model. The average number of commuters ranges from 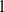 to 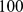. As we expect, no significant effect on the outbreak size distribution can be seen, even for 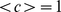. It validates our assumption of the independence of infectious hosts, necessary for a branching-process formulation, as the simulations closely match the analytical predictions. It is noteworthy here that only the biology of the novel pathogen determines the emergence process, as outbreak sizes group according to the intermediate average reproductive number 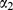. The minimal deviation for 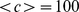 commuters is based on the fact that the effective village size is only 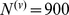 due to the absent commuters.

**Figure 4:**
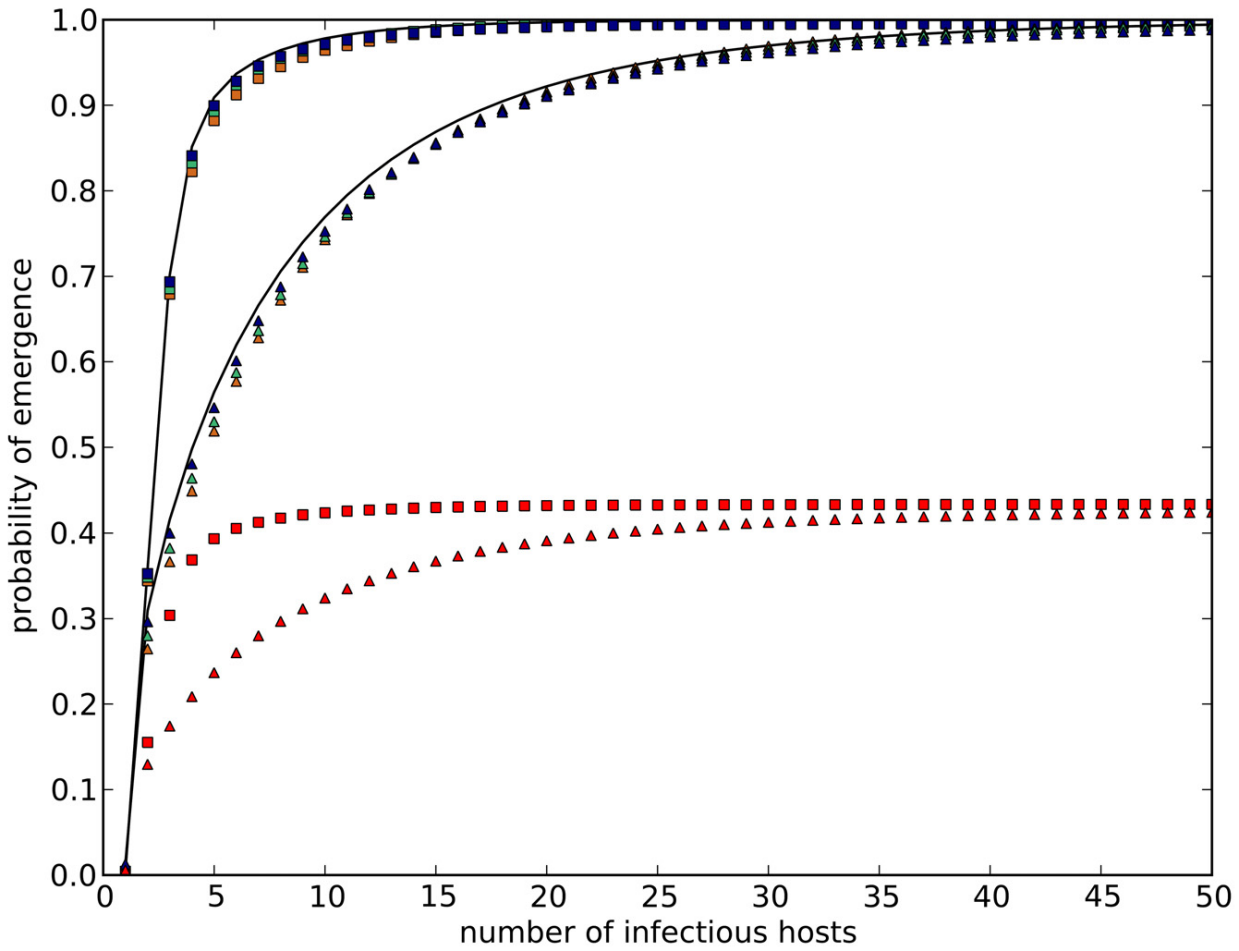
Probability of emergence in the city with short-term commuting. The probability is a function of the overall number of infectious hosts in the village-city model. The solid black lines represent the analytical results for an infinite population without spatial heterogeneous structure. Data points are the average probability of 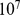 simulations. Squares represent the punctuated route (I), and triangles the gradual route (II), both with the mutation probability 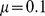. As with the outbreak size distribution, probabilities group according to the route of adaptation instead of connection strength in number of commuters. However, the probability of emergence does not converge to 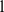 for 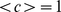 commuters, revealing the effect of spatial heterogeneity when the number of commuters is small relative to the average reproductive number for the well-adapted strain.

In Figure 4, we extract the probability of emergence per introduction given a certain number already infected. These data are easily calculated using the outbreak size distribution and the probability of emergence per introduction. Assume an introduction has caused 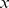 infectious hosts already. The probability of extinction is the cumulative probability of getting an outbreak size equal to or larger than 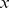, renormalized by all possible outcomes (extinctions and emergences) once 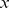 hosts are infectious. This yields the probability of emergence per number of infected. In Figure 4, the effect of spatial heterogeneity can be seen directly. For 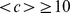, the village-city simulations agree very well with the analytical results assuming a single, infinite population. But for 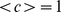, the probability of emergence converges to approximately 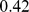.

While Figure 4 shows the probability of emergence as a function of the number infected, the actual outcome is highly unpredictable even if the probability of an event is known as the average waiting time to an emergence shows (see equations 4 and 5). It can be generalized for the probability of emergence given 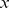 infectious hosts. For example, the probability of emergence given five infectious hosts in the gradual route (II) is 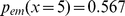. It follows on average every 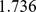 times this happens an emergence will happen. The standard deviation is 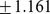, which leads to the conclusion that even if the probability is known, it is inherently unpredictable when this will actually lead to an emergence.

### Spatial Homogeneity Coefficient

**Figure 5:**
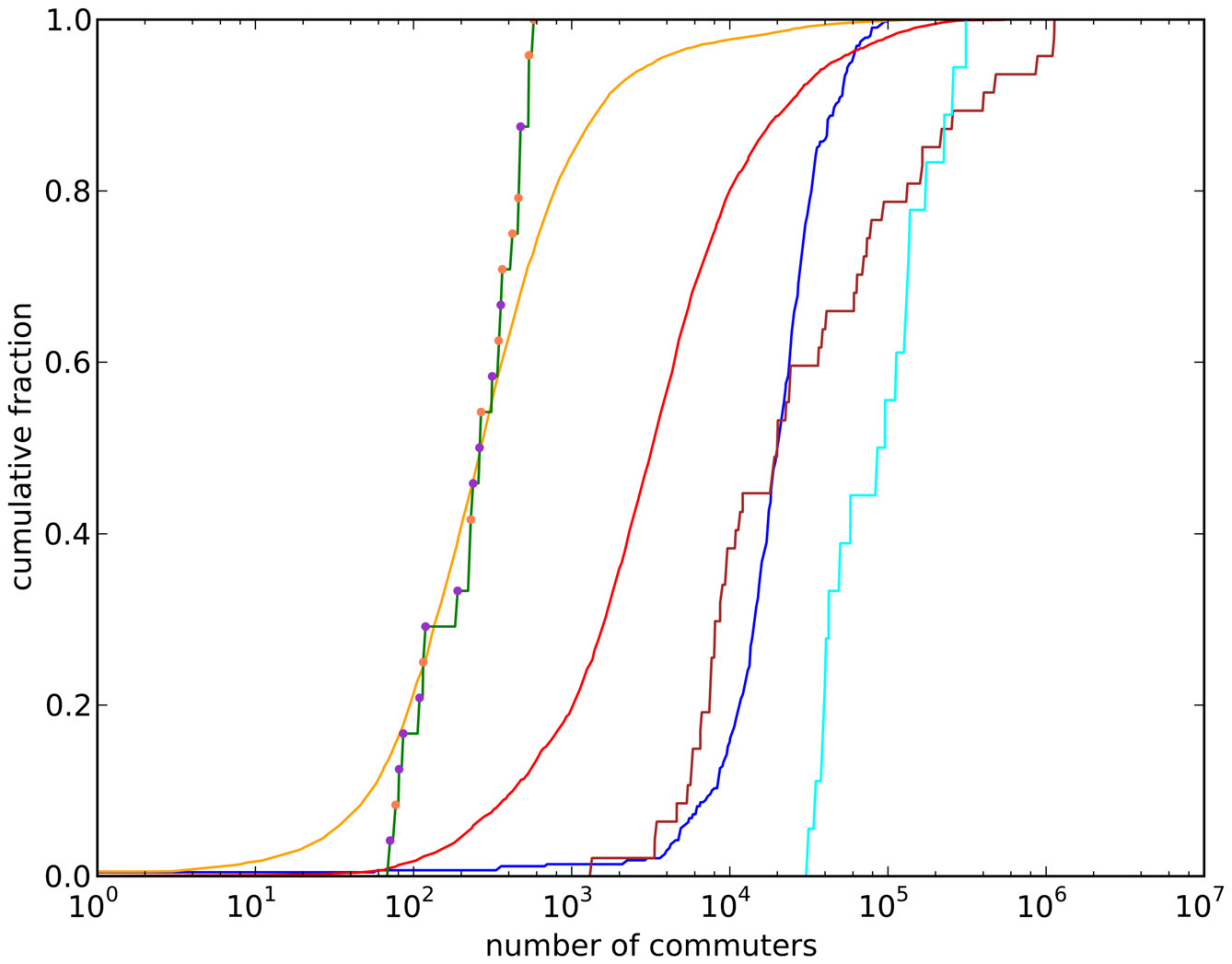
Data of commuting patterns in different parts of the world. Shown is the cumulative fraction of all communities with equal or less than the specified number of commuters. The data was mostly collected by the National Statistical Offices of the respective countries. The gold line represents commuting data from Brazil [21], the red line data from the USA [22], the blue line data from the UK [23], the brown line data from Japan [25], the cyan line data from Hong Kong [24], and the green line data from two independent sources. The green line has orange [27] and pink data points [26], corresponding to its data sources. Our data represents the commuting flows between administrative units. The definition of administrative units varies highly between countries. For example, the US data is on a granularity of 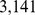 counties, while the data from Japan is based on its 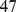 prefectures. However, heterogeneity can also be found within countries datasets. The Brazilian data is on a level of 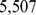 municipalities with resident sizes ranging from 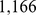 to 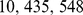.

This confirms that a pathogen needs a sufficient connection between communities to emerge, despite its ability to cause outbreaks, regardless of the spatial structure. Hence we expect the existence of a threshold where spatial heterogeneity effectively does not matter any more. Previous research has shown that the effect of heterogeneity in spatially structured population models depends on the interconnectivity with a threshold effectively allowing the pathogen to spread between communities [18]–[20]. Our approach allows new insights, as we do not need to specify the actual number of infectious hosts migrating to a new community. We measure connectivity between communities in terms of the average number of commuters 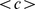 for which rich empirical datasets can be found. Figure 5 presents illustrative examples of empirical data of commuting patterns in different parts of the world. Most data has been collected by Offices of Statistics of five countries on three continents [21]–[25]. A further, two independent studies have been used to estimate commuting patterns of towns in Indonesia [26] and China [27].

We next attempt to quantify the regimes in 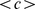 for which spatial heterogeneity may be neglected. We approach this question using a simple analytical derivation for the effect of spatial heterogeneity, which considers only the adapted strain. Assume a connected community such as the village in our village-city model, with a fully adapted pathogen introduced into the village. Given an emergence and epidemic in the village, the probability that this causes an emergence and epidemic in the city is
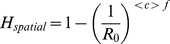
(12)Therefore, 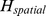 is a spatial homogeneity coefficient, measuring the impact of spatial heterogeneity on an emergence process. It ranges from 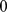, leading to two isolated communities, to 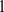, effectively removing any spatial heterogeneity and forming one homogeneous population. 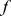 is the fraction of the village residents becoming infectious. It can be derived using [28]

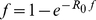
(13)The spatial homogeneity coefficient depends only on the connection strength expressed in commuters 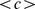 and the average reproductive number 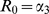 of the fully adapted strain. Though it only considers the fully adapted strain, we expect this coefficient to be a good approximation for a multi-strain model as the vast majority of infectious hosts will transmit the fully adapted strain in the case of an emergence.

**Figure 6:**
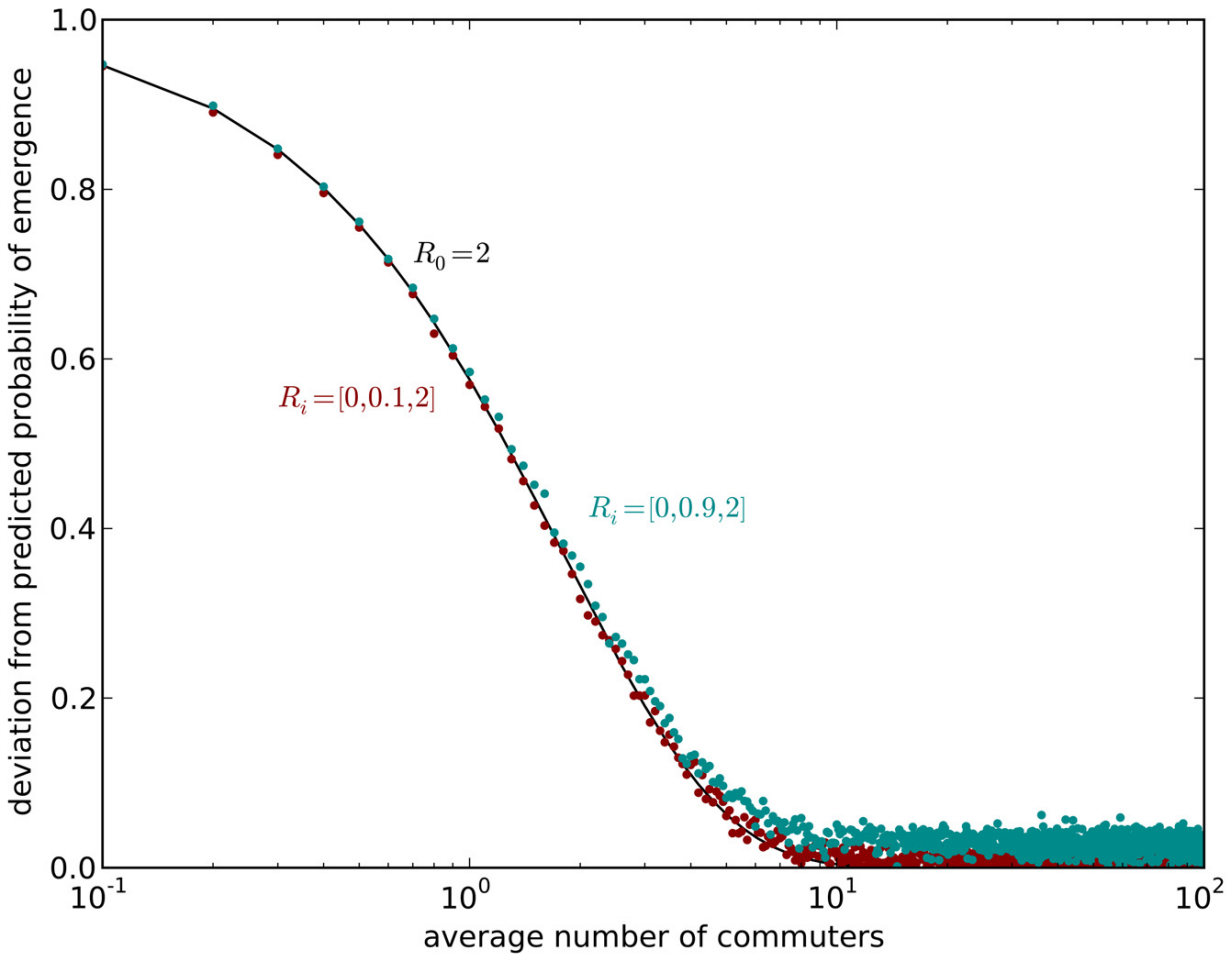
Deviation between simulated and analytical predicted probability of emergence. The deviation is a function of the average number of commuters 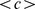. The deviation is defined as 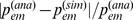. The analytical probability of emergence 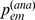 is for an infinite ulation without spatial structure. The simulated probability of emergence 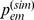 is for short-term commuting with the blue data points representing the gradual route (II) of adaptation, and the orange data points representing the punctuated route (I). The solid black line is 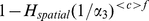, as defined in equation 12 in the main text. It is the analytical expected deviation for spatial heterogeneity as a function of the spatial homogeneity coefficient. The simulations agree very well with the analytical expected deviation. The gradual route (II) is slightly more off from the theoretical prediction as a result of the small but significant number of infected with the intermediate strain. Effectively, it lowers the number of commuters infected with the fully adapted strain and therefore the probability of transmission from the village to the city. Nevertheless, the analytical prediction as well as the simulations show no significant impact of spatial heterogeneity from a critical commuter threshold of 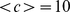.

Figure 6 gives an overview of the influence of spatial heterogeneity as a function of 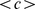. Effectively, spatial heterogeneity is negligible once a critical number of ten commuters connect the two communities. This is a very low threshold, and empirical data shows that the probability of having a community with less than the critical number of ten commuters is approximately 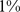 for all our data combined.

As illustrated by the close fit between analytical and numerical results in figure 6, there is only a small error in the analytical expression arising from neglecting infections with mal-adapted strains. This error is greatest for the gradually adapting pathogen, because an intermediate strain with 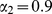 tends to cause larger outbreaks than one with 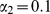. Nevertheless, the deviation remains small.

**Figure 7:**
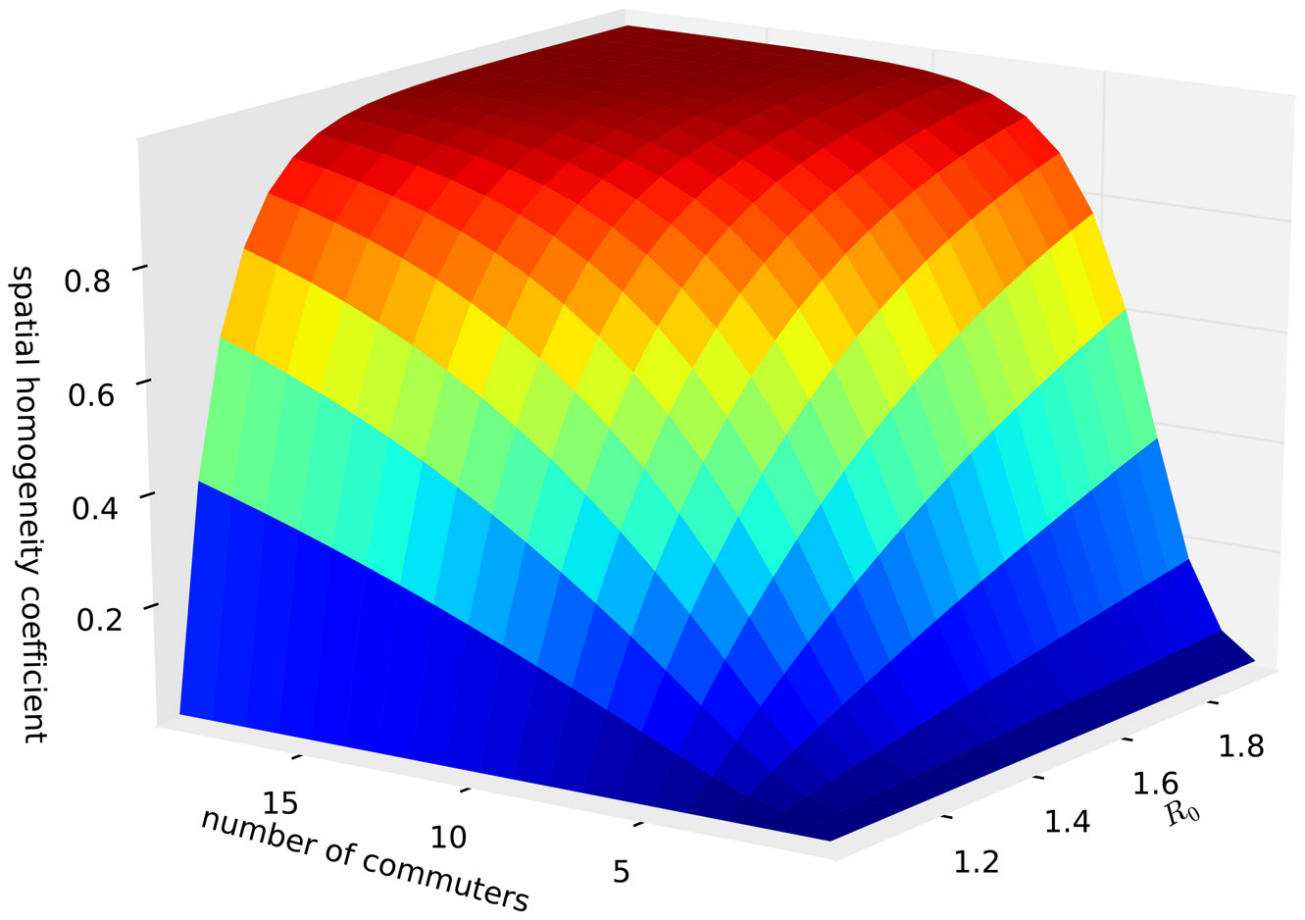
Impact of spatial heterogeneity on disease transmission between communities (I). The impact is measured with the spatial homogeneity coefficient with 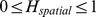. Given 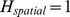 every emergence in the village automatically leads to an emergence in the city, and 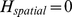 represents no chance of successfully transmitting the pathogen into the city. The figure reveals that spatial structure becomes especially important for small average reproductive numbers. In addition, the average number of commuters needed to show an effect of spatial heterogeneity is surprisingly small.

In light of this agreement, how does the critical average number of commuters vary with the adapted reproductive number? While for 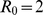 ten average commuters are sufficient to dissolve spatial heterogeneity, this changes dramatically for smaller average reproductive numbers (see Figure 7). If a well-adapted strain is only just pandemic capable (i.e. 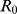 just above 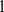) villages with only ten commuters are only 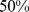 likely to seed an epidemic in their local city, and spatial structure becomes important again. For example, the critical number of commuters is close to 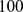 for 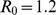. For 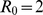 the spatial homogeneity coefficient is approximately 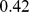 for one commuter. This agrees with what we find in Figure 4 using simulated results.

## Discussion

In this article we first present analytical results to calculate epidemiological parameters of a novel disease, adapting to humans. We explore the influence of spatial host contact structure, and validate our result with stochastic simulations of simple village-city models as an example of interconnected communities within a spatially structured population. Our study reveals that for plausible parameter ranges, spatial heterogeneity only has very limited impact on the probability of emergence, as well as the outbreak size distribution. Neither a change in strength of spatial heterogeneity (e.g. number of commuters), nor in its quality (e.g. short term versus long term commuting) shows a significant influence. Our results suggest that only the most remote rural communities would be subject to epidemiological isolation. In particular, the available empirical data suggests that communities tend to be highly interconnected with relatively high connection strengths. Of course, it is the most remote communities of the world for whom we have the least relevant data. More empirical research on spatial heterogeneity is needed to form a better understanding of its effect, and this need is greatest in developing parts of the world.

In addition, population size becomes an important factor only when that population is relatively small 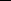 fewer than 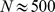 individuals. Only a small number of infectious hosts are actually involved in the emergence process, which relates to the small reservoir of susceptibles needed for a successful emergence. Moreover, biological processes such as the speed of evolution and the adaptive route show a strong influence on the overall emergence process. We show that epidemiological parameters such as the outbreak size group according to the evolutionary route. Previous research has shown the effect of the pathogen's route of evolutionary adaptation and mutation rate on the probability of emergence per introduction [10], [11]. Our theoretical derivation of the probability of emergence extends this and offers the benefit of being analytically solvable for any possible route of adaptation and any mutation probabilities. We note here that previous work has highlighted the role of other, significant types of heterogeneity in emergence of a novel infection. For example, [29] describe the effect of the pathogens life history, such as the length of infection, on the emergence of a novel pathogen. Further, heterogeneity in human-to-human transmission within a population may have an influence on the course and probability of emergence and outbreaks [13], [14], usually lowering the probability of emergence. While we have concentrated here on simple types of spatial heterogeneity, a significant question for future research is the role of mixed heterogeneities, for example spatially structured populations with additional heterogeneity in the human-to-human transmission.

We also find that the waiting time for an outcome of a novel pathogen's introduction is highly unpredictable, even if the probability for such an event is known. Conversely, this means that an estimate of the underlying epidemiological parameters from observed data will be highly uncertain. Unfortunately, a large number of observations will be necessary to achieve confidence in the parameters, and even a large number of introductions gone extinct do not rule out the possibility of emergence for a pathogen. We came to a similar result [11] using a measurement on the upper bound of the probability of emergence. Moreover, the probability of emergence given a certain number of infectious hosts can be surprisingly low. Even a comparatively large number of infectious hosts can end in extinction, especially for low mutation rates and intermediate-stage average reproductive numbers just below one.

Our work has relevance for important public health issues: if a novel disease is detected in a rural setting, and it appears to be spreading, how feasible is it to contain infection by restricting movements to and from the village? Our results suggest that first, an infeasibly tight level of quarantine would be required for any chance of containment, corresponding to enforcing a low level of 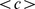 in Figure 6. To all intents and purposes isolation would have to be absolute to be effective. In most circumstances such extreme intervention would not be acceptable. Second, given typical mobility patterns, it is likely that once there is a detectable number of cases in the village, there may already be a significant number of cases outside of it. Therefore quarantining interventions are likely to come too late.

Our work raises important questions for future research: where should surveillance be focused to detect an emergence as early as possible, especially if resources are limited? Given emergence of a novel infection in a rural setting, how much time can we buy through limiting travel to and from major urban centres? These and other questions will undoubtedly benefit from more systematic studies of emergence in the context of population distributions. Nonetheless, theoretical models such as those presented here can offer useful, fundamental insights to guide such studies.

## Supporting Information

Text S1 Supplementary material with figures for ‘Insights into the Evolution and Emergence of a Novel Infectious Disease’.
(0.16 MB PDF)
