# Supplementary material for: Insights into the Evolution and Emergence of a Novel Infectious Disease
Source: PLoS Comput Biol. 2010 Sep 30;6(9):e1000947. doi: 10.1371/journal.pcbi.1000947 (PMC2947978; doi:10.1371/journal.pcbi.1000947)
Supplement: Text S1 — Supplementary material with figures for ‘Insights into the Evolution and Emergence of a Novel Infectious Disease’. (0.16 MB PDF) [file pcbi.1000947.s001.pdf]

A Text S1 - Supplementary Material

A.1 Analytical Derivations

A.1.1 Probability of Emergence

As in [10], our derivations use a multi-type branching formulation to describe the extinction probability in the process of emergence. In general, our n-type branching process is given by n probability generating functions (pgfs)

$$g_i(s_i, \dots, s_n) = \sum_{j_i, \dots, j_n = 0}^{\infty} \phi_i(j_i, \dots, j_n) s_i^{j_i} \dots s_n^{j_n} \tag{1}$$

with $\phi_i(j_i, \dots, j_n)$ being the probability that a single infected host of type i gives rise to j_k secondary infections with $k \in \{i, \dots, n\}$. Hence, for a geometric offspring distribution governed by (2) in the main text, ϕ is

$$\begin{aligned}
\phi_n(j_n) &= p_n^{j_n}\, r_n \\
\phi_{n-1}(j_{n-1}, j_n) &= p_{n-1}^{j_{n-1}}\, r_{n-1} + p_{n-1}^{j_{n-1}}\, m_{n-1}\, p_n^{j_n}\, r_n \\
&\vdots \\
\phi_i(j_i, \dots, j_n) &= p_i^{j_i} \sum_{x=i}^{n} r_x \prod_{y=i+1}^{x} m_{y-1}\, p_y^{j_y}
\end{aligned} \tag{2}$$

Note we define $\prod_a^b \dots = 1$ whenever $a > b$ for all product operators.

The probability of extinction q_i is defined as probability of an introduction which will reach only a finite outbreak size, and go extinct. Hence it is the probability that all secondary cases of every infected host finally lead to extinction. This self-similarity is expressed with

$$g_i(q_i, \dots, q_n) = q_i \tag{3}$$

It is possible to calculate all extinction probabilities, starting with the fully adapted strain. Inserting the probabilities of (2) and taking into account that $p_i, q_i < 1$, the pgfs in (1) become, under the use of the

fixed point relation (3),

$$q_i = \sum_{j_i,\dots,j_n=0}^{\infty} p_i^{j_i}\, q_i^{j_i} \sum_{x=i}^{n} r_x \prod_{y=i+1}^{x} m_{y-1}\, p_y^{j_y}\, q_y^{j_y} \\ = \sum_{x=i}^{n} \frac{r_x}{1-p_i q_i} \prod_{y=i+1}^{x} \frac{m_{y-1}}{1-p_y q_y} \tag{4}$$

It is possible to find a closed form for the probabilities of extinction with a few lines of derivations. If $p_i = 0$, equation (4) becomes

$$q_i = \sum_{x=i}^{n} r_x \prod_{y=i+1}^{x} \frac{m_{y-1}}{1-p_y q_y} \tag{5}$$

If $p_i > 0$, equation (4) becomes

$$q_i = \frac{1}{2p_i} - \sqrt{\frac{1}{4p_i^2} - \sum_{x=i}^{n} \frac{r_x}{p_i} \prod_{y=i+1}^{x} \frac{m_{y-1}}{1-p_y q_y}} \tag{6}$$

Note that q_i with $p_i > 0$ has two analytical solutions with $q_i = 1$ as trivial one. Only the solution shown in (6) needs to be explicitly calculated.

If a wildtype strain is introduced at each introduction, the probability of extinction becomes q_0. The probability of emergence per introduction p_{em} is one minus the probability of extinction

$$p_{em} = 1 - \begin{cases} \sum_{x=1}^{n} r_x \prod_{y=2}^{x} \frac{m_{y-1}}{1-p_y q_y} & \text{if } p_1 = \alpha_1 = 0 \\ \frac{1}{2p_1} - \sqrt{\frac{1}{4p_1^2} - \sum_{x=1}^{n} \frac{r_x}{p_1} \prod_{y=2}^{x} \frac{m_{y-1}}{1-p_y q_y}} & \text{otherwise} \end{cases} \tag{7}$$

This expression can be solved analytically for all possible routes of adaptation.

A.1.2 Outbreak Size Distribution

An emerging disease in a population can be modelled as individual particles with new offspring, as done in the branching process calculations above. State-space models are another representation. These focus not on individual hosts, but the "states" in which these hosts are. Instead of having X hosts moving between the states of being susceptible, infected, or recovered, the system gets transformed into moving through Y states corresponding to the number of susceptibles, infected, or recovered hosts. The probabilities to infect, mutate, or recover are constant over time for each host, and so are the transition rates between

the states.

Let the system be in state $I_{i,t}$, corresponding to the number of infectious hosts with R_i at transition t. The number of transitions t is similar to a time as it goes only in one direction. Each change of state, which means every infection, mutation, or recovery, is a single transition. Hence the rate of entering the state $I_{i,t+1}+1$ is equal to the probability of infection plus the probability of mutation, p_i and m_{i-1}. The transition into state $I_{i,t+1}-1$ can happen either by mutation or recovery. Hence the according transition rates are m_i and r_i. This leads to the following Master-Equation for $I_{i,t}>1$ with $\rho_i(I_{i,t},t)$ as probability of being in state $I_{i,t}$

$$\rho_i(I_{i,t},t) = (p_i + m_{i-1})\ \rho_i(I_{i,t}-1,t-1) + (m_i + r_i)\ \rho_i(I_{i,t}+1,t-1) \tag{8}$$

Otherwise it is for $I_{i,t}=1$

$$\rho_i(1,t) = m_{i-1}\ \rho_i(0,t-1) + (m_i + r_i)\ \rho_i(2,t-1) \tag{9}$$

And for $I_{i,t}=0$

$$\rho_i(0,t) = \rho_i(0,t-1) + (m_i + r_i)\ \rho_i(1,t-1) \tag{10}$$

The initial condition at $t=0$ is all probabilities are 0 except $\rho_i(I_{i,0},0)=1$. $I_{i,0}$ is the number of infected hosts to start with, also known as "patient zeros". Going back to the branching process interpretation, each infected host is assumed independent from each other. The same idea is true in the Master-Equation interpretation. The time of the transition through mutation is irrelevant, only the transition event itself counts. Hence it is possible to sum up all the mutation events from strain $i-1$ to i and add these number to the initial number of infected hosts with strain i at $t=0$.

In addition, the end condition is also known. Let strain i go extinct at transition $t=T$. Hence the system must have been in state 1 at transition $t=T-1$ and

$$x_i = \frac{T - I_{i,0}}{2} \tag{11}$$

of the past transition events must have been infections. x_i is also the outbreak size of secondary infected

hosts. Accordingly the number of transitions for a given outbreak size is

$$T = 2x_i + I_{i,0} \tag{12}$$

Equation (12) reveals a direct relation between the number of transitions and the number of secondary infections. It enables us to derive the outbreak size probability $g_i(I_{i,0}, x_i)$, as it is possible to derive $\rho_i(1,t)$ for every t with the initial condition $I_{i,0}$ and the Master-Equations (8), (9), and (10). In general, the outbreak size probability is

$$g_i(I_{i,0}, x_i) = A\ p_i^{x_i}\ \ (r_i + m_i)^{x_i + I_{i,0}} \tag{13}$$

with

$$A = \begin{cases} 0 & \text{if } I_{i,0} = 0 \\ 1 & \text{if } x_i = I_{i,0} \\ \binom{2x_i + I_{i,0} - 1}{2x_i + I_{i,0} - 1} - \binom{2x_i + I_{i,0} - 1}{2x_i + I_{i,0}} & \text{otherwise} \end{cases} \tag{14}$$

Note that x_i is only the number of new infected hosts in i, not the mutated ones $I_{i,0}$. It is necessary to subtract these to prevent over-counting of infected. The derivations can be done analogous to the ones above to count $I_{i,0}$ in at strain i instead of $i-1$.

The number of initially infected hosts $I_{i,0}$ depends on x_{i-1} and the $I_{i-1,0}$ as only infected host with strain $i-1$ can possibly mutate into strain i. The probability of mutation for each infected host is $\frac{\mu_{i-1}}{\mu_{i-1}+1} = \frac{m_{i-1}}{m_{i-1}+r_{i-1}}$. As the number of mutations is binomial distributed, the probability of this initial condition is

$$\zeta_i(I_{i,0}) = g_{i-1}(I_{i-1,0}, x_{i-1}) \binom{I_{i-1,0} + x_{i-1}}{I_{i,0}} \left(\frac{m_{i-1}}{m_{i-1} + r_{i-1}}\right)^{I_{i,0}} \left(\frac{r_{i-1}}{m_{i-1} + r_{i-1}}\right)^{I_{i-1,0} + x_{i-1} - I_{i,0}} \tag{15}$$

It is possible to calculate the distribution of each strain by recursive derivation, starting with the wildtype strain. Furthermore the overall outbreak size probability can be derived using conditional probabilities

$$p_{out}(X) = g_1(I_{1,0}, x_1) \sum_{I_{2,0},\ldots,I_{n,0}=0}^{I_{1,0}+x_1,\ldots,I_{n-1,0}+x_{n-1}} \prod_{i=2}^{n} \zeta_i(I_{i,0})\ g_i(I_{i,0}, x_i) \tag{16}$$

with $X = I_{1,0} + \sum_{i=1}^{n} x_i$ as overall outbreak size. In addition, it is straight forward to derive the probability

of emergence given p_{out}

$$p_{em} = 1 - \sum_{X=1}^{\infty} p_{out}(X) \tag{17}$$

While equation (7) is the fastest way to compute p_{em}, equation (17) can be used as well.

A.1.3 Waiting Time to Emergence

Let the probability of emergence per introduction be p_{em} with $0 \leq p_{em} \leq 1$. Hence the probability of having an emergence after M introductions is

$$\begin{aligned} P_{em}(M) &= (1 - p_{em})^M \, p_{em} \\ &= q_{ext}^M \, p_{em} \end{aligned} \tag{18}$$

with $q_{ext} = 1 - p_{em}$ as probability of extinction per introduction. A convenient way to handle probability distributions is with pgfs

$$\begin{aligned} G_M(z) &= \sum_{M=0}^{\infty} P_{em}(M) z^M \\ &= p_{em} \sum_{M=0}^{\infty} (q_{ext}\, z)^M \\ &= \frac{p_{em}}{1 - q_{ext}\, z} \end{aligned} \tag{19}$$

with $|z| < \frac{1}{q_{ext}}$.

Using known characteristic of pgfs, it is possible to calculate the expected number of introductions

$$\begin{aligned} \langle M \rangle = G'_M(1) &= \left[\frac{d}{dM} G_M(z) \right]_{z=1} \\ &= p_{em} \sum_{M=0}^{\infty} M q_{ext}^M \\ &= p_{em} \frac{1 - p_{em}}{p_{em}^2} \\ &= \frac{1}{p_{em}} - 1 \end{aligned} \tag{20}$$

Note that this is the average number of introductions without an emergence, as defined in (18). The average number of introductions needed to get an emergence event is $\langle M \rangle + 1$.

In addition, the variance can be obtained in a similar way

$$\begin{aligned}
\text{var}(M) =& G''_M(1) + G'_M(1) - [G'_M(1)]^2 \\
=& \left[p_{em} \sum_{M=0}^{\infty} M(M-1) q_{ext}^M z^{M-2}\right]_{z=1} + \left[p_{em} \sum_{M=0}^{\infty} M q_{ext}^M z^{M-1}\right]_{z=1} \\
&- \left(\left[p_{em} \sum_{M=0}^{\infty} M q_{ext}^M z^{M-1}\right]_{z=1}\right)^2 \\
=& \frac{1}{p_{em}^2} - \frac{1}{p_{em}}
\end{aligned} \tag{21}$$

The variance is very large, and therefore the peak around the expected value very wide. The standard deviation is of the same magnitude as the mean. This makes the number of needed introductions highly unpredictable.

A.2 Spatial Heterogeneity on Larger Timescales

As for commuting on a daily basis, we focus on a simple village - city model to approximate the spatial heterogeneity in rural areas only weakly connected to bigger cities. As before, we have a wildtype pathogen capable of acquiring adaptations for human transmission. Assume $N^{(v)} = 1000$ villagers, and an infinite number of hosts in the city. Moreover, assume a commuting rate of χ between city and village. As discussed in the main text, we assume here that commuting is on a timescale longer than the infectious period, but may be temporary. Thus we assume that the return rate is the same as the outgoing rate, in order to maintain a constant number of hosts in the village. Figure 1 shows a schematic representation of the model.

For the village, we write: $S^{(v)}$ for the number of susceptibles, and $I_i^{(v)}$ for the numbers of infected with strain i. Correspondingly, $I_i^{(c)}$ is the number infected with type i in the city. Then, assuming that all strains have the same mean infectious period, and normalising continuous time with respect to this

mean infectious period, an ODE model for this system reads

$$\begin{aligned}\frac{dS^{(v)}}{dt} &= -\sum_{i=1}^{3}\alpha_i I_i^{(v)}\frac{S^{(v)}}{N^{(v)}} + \chi(N^{(v)} - S^{(v)})\\ \frac{dI_i^{(v)}}{dt} &= \alpha_i I_i^{(v)}\frac{S^{(v)}}{N^{(v)}} + \mu_{i-1}I_{i-1}^{(v)} - (\chi + \mu_i + 1)\, I_i^{(v)}\\ \frac{dI_i^{(c)}}{dt} &= \alpha_i\left(I_i^{(c)} + I_i^{(v)}\right) + \mu_{i-1}I_{i-1}^{(c)} - (\chi + \mu_i + 1)\, I_i^{(c)}\end{aligned} \tag{22}$$

where μ_i and α_i are, as before, respectively the mutation number and basic reproductive number associated with strain i. Our initial conditions are $I_1^{(v)} = 1$, and all other infected numbers zero, which means the infection is seeded with a wildtype strain in the village. An emergence is defined as before (100 infected with the fully adapted strain in the city).

Figure 2 shows the outbreak size distribution of our long-term migration model, compared with the analytical results for one infinite population. The number of commuters varies between 10 and 100. Even for the smallest average number of commuters the simulations agree very well with the analytical derivations, a result consistent with that for the short term commuting model discussed in the main text.

Figure 3 shows the probability of emergence as a function of the infectious host number. Again, the analytical derivations show very good agreement with the simulations, neglecting an influence of spatial heterogeneity, and revealing a grouping according to the biological adaptation process of the novel pathogen. Note that Figure 3 also reveals the possibility of an emergence in the village without further sustained transmission in the city for $\langle c\rangle = 10$ as the probability of emergence does not reach 1. Nevertheless, this is an very unlikely scenario with a probability of less than 0.02 at 100 infected hosts.

Interestingly, five infectious hosts or more are need in the gradual route (II) before emergence becomes more likely than extinction (see Figure 3). In the punctuated route (I), this becomes three or more infected, caused by the very low probability of generating secondary infected with an average reproductive number of $\alpha_2 = 0.1$. Secondary infections are most likely to occur with the fully adapted strain only.

A.3 Figures

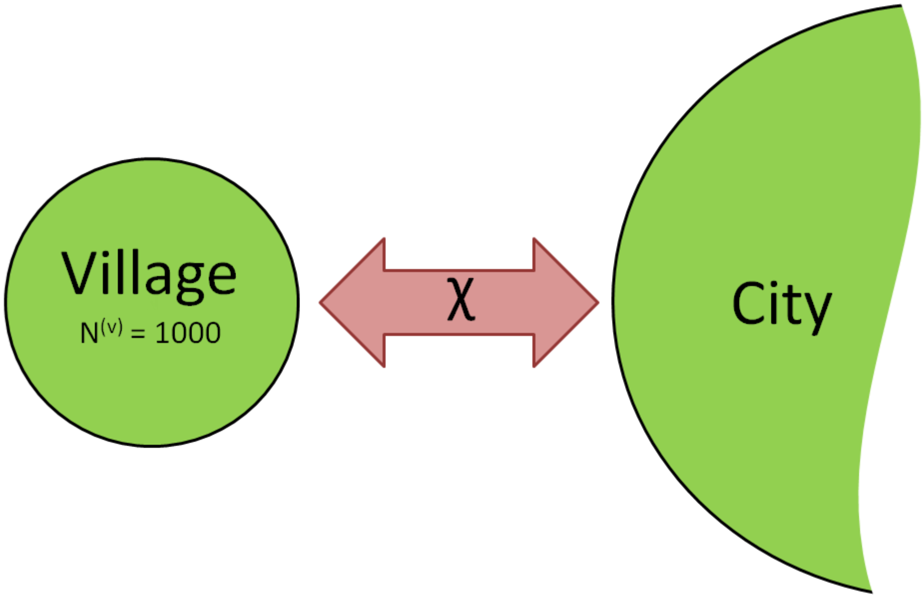

Figure 1. Schematic representation of the long-term commuting model. $N^{(v)}$ is the number of hosts in the village and χ the migration rate. Note the number of commuters from the village to the city is a function of the village size and the migration rate. The city has an infinite number of inhabitants.

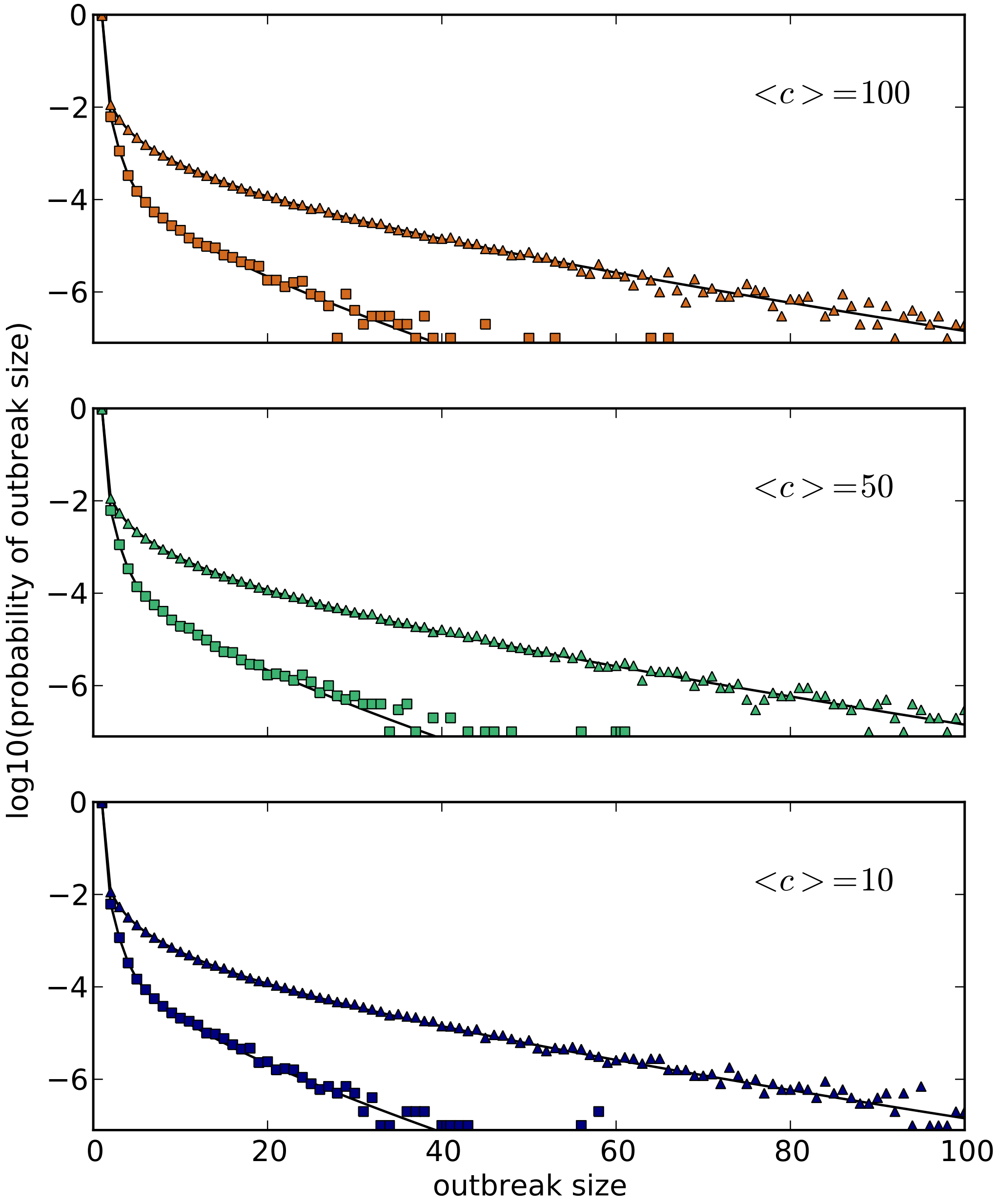

Figure 2. Outbreak size distribution of the village-city model with long-term commuting. The solid black lines represent the analytical results for an infinite population without spatial heterogeneous structure. Data points are the average probability of 10^7 simulations. Squares represent the punctuated route (I), and triangles the gradual route (II), both with the mutation probability $\mu = 0.1$ (see main article). As for homogeneous populations, the outbreak size distributions group according to the evolutionary route of adaptation. The average number of commuters, $\langle c \rangle$, does not have an influence as all simulations do not show a significant variation from the analytical results.

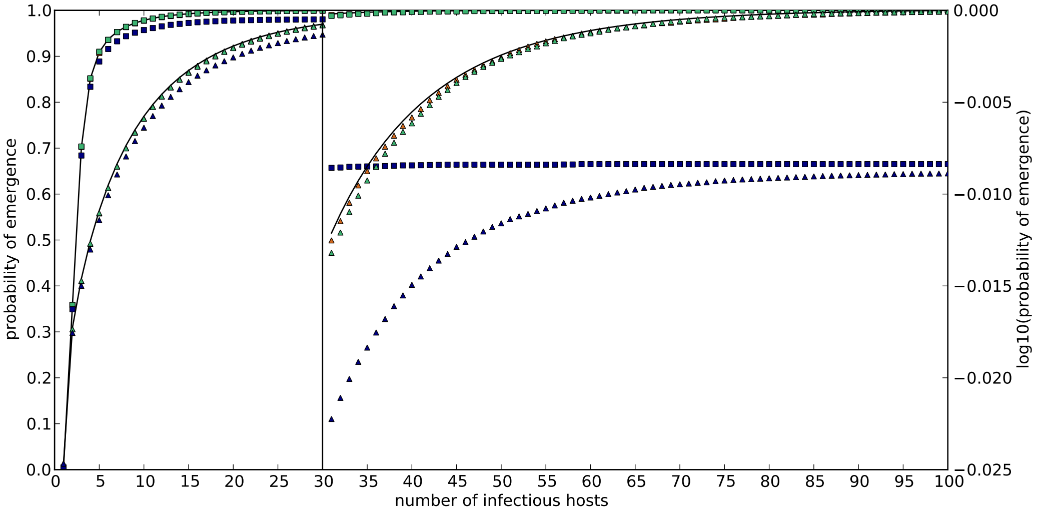

Figure 3. Probability of emergence in the city with long-term commuting. The probability is a function of the overall number of infectious hosts in the village-city model. The solid black lines represent the analytical results for an infinite population without spatial heterogeneous structure. Data points are the average probability of 10^7 simulations. Squares represent the punctuated route (I), and triangles the gradual route (II), both with the mutation probability $\mu = 0.1$ (see main article). The orange colour represent an average number of commuters $\langle c \rangle = 10$, the blue colour $\langle c \rangle = 50$, and the red colour $\langle c \rangle = 100$. The plot is dived at 30 infectious hosts into a linear probability scale (left), and a log10 probability scale (right). As with the outbreak size distribution, probabilities group according to the route of adaptation instead of connection strength in number of commuters. However, the probability of emergence does not converge to 1 for $\langle c \rangle = 10$ commuters. Here the effect of spatial heterogeneity is very small but visible as a small number of commuters can lead to an emergence in the village without further, sustained transmission to and within the city.
